# The Epidemiology of Hypertension in Uganda: Findings from the National Non-Communicable Diseases Risk Factor Survey

**DOI:** 10.1371/journal.pone.0138991

**Published:** 2015-09-25

**Authors:** David Guwatudde, Gerald Mutungi, Ronald Wesonga, Richard Kajjura, Hafisa Kasule, James Muwonge, Vincent Ssenono, Silver K. Bahendeka

**Affiliations:** 1 Department of Epidemiology & Biostatistics, School of Public Health, Makerere University College of Health Sciences, Kampala, Uganda; 2 Control of Non-Communicable Diseases Desk, Ministry of Health, Kampala, Uganda; 3 School of Statistics and Planning, Makerere University College of Business and Management Sciences, Kampala, Uganda; 4 Department of Community Health and Behavioral Sciences, School of Public Health, Makerere University College of Health Sciences, Kampala, Uganda; 5 World Health Organization, Uganda Country Office, Kampala, Uganda; 6 Division of Socioeconomic Surveys, Uganda Bureau of Statistics, Kampala, Uganda; 7 Department of Internal Medicine, Nsambya Hospital, Kampala, Uganda; National Cardiovascular Center Hospital, JAPAN

## Abstract

**Background:**

Hypertension is an important contributor to global burden of disease and mortality, and is a growing public health problem in sub-Saharan Africa. However, most sub-Saharan African countries lack detailed countrywide data on hypertension and other non-communicable diseases (NCD) risk factors that would provide benchmark information for design of appropriate interventions. We analyzed blood pressure data from Uganda’s nationwide NCD risk factor survey conducted in 2014, to describe the prevalence and distribution of hypertension in the Ugandan population, and to identify the associated factors.

**Methods:**

The NCD risk factor survey drew a countrywide sample stratified by the four regions of the country, and with separate estimates for rural and urban areas. The World Health Organization’s STEPs tool was used to collect data on demographic and behavioral characteristics, and physical and biochemical measurements. Prevalence rate ratios (PRR) using modified Poison regression modelling was used to identify factors associated with hypertension.

**Results:**

Of the 3906 participants, 1033 were classified as hypertensive, giving an overall prevalence of 26.4%. Prevalence was highest in the central region at 28.5%, followed by the eastern region at 26.4%, western region at 26.3%, and northern region at 23.3%. Prevalence in urban areas was 28.9%, and 25.8% in rural areas. The differences between regions, and between rural-urban areas were not statistically significant. Only 7.7% of participants with hypertension were aware of their high blood pressure. The prevalence of pre-hypertension was also high at 36.9%. The only modifiable factor found to be associated with hypertension was higher body mass index (BMI). Compared to participants with BMI less than 25 kg/m^2^, prevalence was significantly higher among participants with BMI between 25 to 29.9 kg/m^2^ with an adjusted PRR = 1.46 [95% CI = 1.25–1.71], and even higher among obese participants (BMI ≥ 30 kg/m^2^) with an adjusted PRR = 1.60 [95% CI = 1.29–1.99]. The un-modifiable factor found to be associated with hypertension was older age with an adjusted PRR of 1.02 [95% CI = 1.02–1.03] per yearly increase in age.

**Conclusions:**

The prevalence of hypertension in Uganda is high, with no significant differences in distribution by geographical location. Only 7.7% of persons with hypertension were aware of their hypertension, indicating a high burden of undiagnosed and un-controlled high blood pressure. Thus a big percentage of persons with hypertension are at high risk of hypertension-related cardiovascular NCDs.

## Introduction

Hypertension is the most common cardiovascular disorder, affecting approximately one billion people globally, and is among the leading contributors to the global burden of disease and premature death, accounting for approximately 9.4 million deaths annually [1–5]. In 2000 alone, there was an estimated 972 million people with hypertension, 65% of whom lived in the developing world. The effects of hypertension if not controlled are devastating, and may lead to a number of non-communicable diseases (NCDs) including stroke, myocardial infarction, cardiac failure, and renal failure among others [6]. In sub-Saharan Africa (SSA), many countries still lack detailed basic data on the prevalence and the determinants of hypertension [3]. However the World Health Organization (WHO) projects the number of hypertension cases in SSA to increase from an estimated 80 million in 2000 to 150 million in 2025 [7].

In Uganda, the few studies conducted to estimate the prevalence of hypertension have been in small geographical areas and therefore have not sufficiently clarified the prevalence of hypertension countrywide. The studies include three conducted in the western districts of Rukunguri [8], Mbarara [9] and Karese [10], another conducted in the south western district of Masaka [11], and recently a study conducted in the central districts of Buikwe and Mukono [12]. No national survey had ever been conducted to provide countrywide prevalence of the various risk factors for NCDs, including hypertension. In 2014, Uganda’s Ministry of Health commissioned the first countrywide NCD risk factor survey to provide baseline estimates of the prevalence of common risk factors for NCDs in Uganda. We analyzed blood pressure data from this survey to describe the prevalence of hypertension and its distribution in the Ugandan population, as well as identify the factors associated with hypertension.

## Materials and Methods

A cross-sectional study design was used to conduct the survey between March and July 2014. The survey used the World Health Organization’s (WHO) STEPwise approach to surveillance, a standardized method of analyzing risk factors for NCDs. The STEPwise approach is a sequential process starting with collecting data on key risk factors using a questionnaire (STEP 1), followed by physical measurements (STEP 2), and finally collection of blood samples for biochemical assessments (STEP 3) [13].

### Study population and sampling

Uganda has a total population of 34.9 million people, approximately 43% of which are adults aged 18 years or older [14]. The survey covered the whole country, and a three stage sampling design was used to select participants. The sampling procedure utilized the Uganda Bureau of Statistics (UBOS) master sampling frame of Enumeration Areas (EAs) that had just been demarcated throughout the country in preparation for the 2014 population and housing census. Each EA included 150–200 households. In the first stage, a random sample of 350 out of 78,950 EAs was selected with selection probability proportional to the size (PPS) of the number of households in the EAs. The EAs were stratified across the four regions of Uganda namely: Central, Eastern, Northern and Western region; and were selected with separate estimates for rural and urban areas. Urban areas were defined as EAs within government designated urban areas, or those within other geographic divisions with population density of more than 1000 per square kilometer.

After selecting the 350 EAs, trained teams of UBOS staff were dispatched throughout the country to list the households and their household heads within the 350 EAs. A household was defined as a group of individuals that usually shared meals together, and had a household head who usually made major decisions for the household. In the second stage of sampling, 14 households were randomly selected from the listed households in each of the sampled EAs.

Research Assistants (RA) that had received a five-day training on procedures and administration of the STEPs tool, enumerated eligible household members who were recorded in Personal Digital Assistants (PDA), which was then used to randomly select one subject for inclusion in the survey giving a total sample of 4900. Eligible subjects were household members aged 18 to 69 years, who had resided in the sampled households for at least six months preceding the date of interview.

Data were collected using the standard WHO STEPs tool for NCD risk factor surveillance [13]. Trained interviewers administered the various sections of the modular tool using PDAs. The tool was translated to six commonly spoken local languages in Uganda, namely Runyoro-Rutoro, Luo, Ateso, Luganda, Lugbara and Runyankore-Rukiga. These languages have previously been successfully used by UBOS in the Uganda National Household Surveys with excellent national coverage. Participants were given the option of being interviewed in any one of the six languages of their choice.

### Measurements

In STEP 1, the tool collected demographic, socio-economic and behavioral characteristics (including tobacco use, alcohol use, fruit and vegetable consumption, and physical activity).

Current alcohol users were participants who reported having consumed any type of alcohol in the past 30 days preceding the survey. Alcohol consumption was measured in terms of equivalent standard drinks of alcohol consumed. The World Health Organization (WHO) defines 1 standard alcoholic drink as any alcohol drink that contains 10 grams of pure alcohol [15,16]. The following measures were used as equivalent to 1 standard alcoholic drink: a) A 285 ml bottle or can of beer, b) a 100 ml glass of wine (factory distilled or locally brewed), and c) a 30 ml glass of a spirit or gin (factory distilled or locally brewed) [17].

Alcohol users were categorized into heavy, medium, or low users. Heavy alcohol users were participants reporting binge drinking in the past 30 days (an equivalent of more than 6 standard drinks of alcohol in one sitting; and/or consuming an equivalent of more than 6 standard drinks of alcohol on average per occasion among men, or an equivalent of more than 4 standard drinks of alcohol on average per occasion among women. Medium alcohol users were participants reporting consuming an equivalent of 4 to 6 standard drinks of alcohol on average per occasion among men, or 2 to 4 standard drinks of alcohol on average per occasion among women. Low alcohol users were participants reporting consuming an equivalent of less than 4 standard drinks of alcohol on average per occasion among men, or less than 2 standard drinks of alcohol on average per occasion among women [17].

In STEP 2 physical measurements were made that included weight, height waist and hip circumferences, and blood pressure. Blood pressure measurements were taken on the left arm with the participant in the sitting position using battery powered digital blood pressure machine (Boso Medicus Uno**®**). Three readings were taken 3–5 minutes apart. Heart rate reading was also recorded. Standing height was measured with a laser stadiometer incorporated in the compact SECA 877 that includes a pre-calibrated digital weighing scale. In case of laser sensor stadiometer failure, height was manually measured with the participant standing upright against a wall on which a height mark was made and a tape measure used to derive the height. Measurements were taken with the participant in barefoot, standing with the back and head against the wall, with heels together. The participant was asked to stretch to the fullest. Weight measurements were taken using a pre-calibrated digital weighing scale (SECA 877). Height was measured without shoes, to the nearest centimetre. Weight was measured with the participant in light clothing and without footwear using a weighing scale to the nearest tenth of a kilogram.

After administering the questionnaire and taking the physical measurements, interviewers requested participants to converge at pre-arranged location the following morning so that a blood sample could be obtained to conduct the biochemical measurements (STEP 3). Participants were instructed to fast overnight and no exercise or smoking in the morning, in preparation for obtaining a blood sample to conduct the biochemical measurements. The following morning participants in each EA converged at the agreed location where finger blood samples for biochemical tests were taken. Only participants reporting compliance with an overnight 8-hr fast, no exercise or smoking that morning were eligible for finger prick blood sample collection. Non-compliers were rescheduled to future a date where possible; otherwise this procedure was omitted in such participants. Total cholesterol, High Density Lipoprotein Cholesterol (HDL-C) and fasting plasma glucose were measured using CardioChek® PA meter.

### Ethics

The conduct of the survey was approved by the Institutional Review Committee of Nsambya Hospital, Kampala, Uganda, and registered by the Uganda National Council for Science and Technology (UNCST). Written informed consent was obtained from eligible subjects before enrollment in the study. Participants with at least two systolic blood pressure readings of at least 120 mm Hg, and/or diastolic blood pressure of at least 80mm Hg, and/or with fasting plasma glucose of at least 6.1 mmol/L, and were not already on treatment for hypertension and/or diabetes, were advised to as soon as possible report to the nearest government owned health facility for further evaluation.

### Statistical Analysis

The average of the last two blood pressure readings was used in analysis. A participant was classified as hypertensive if their average systolic blood pressure (SBP) was at least 140 mm Hg, and/or their average diastolic blood pressure (DBP) was at least 90 mm Hg, or if they reported being on regular anti-hypertensive therapy. A participant was classified as pre-hypertensive if their average SBP was between 120 and 139 mm Hg (inclusive), and/or their DBP was between 80 and 89 mm Hg (inclusive), and not on any anti-hypertensive therapy [18,19].

The prevalence of pre-hypertension and hypertension were calculated as the percentage of participants classified as pre-hypertensive or hypertensive (respectively). To identify factors associated with hypertension, weighted modified Poisson regression modeling with robust variance [20,21] was used to estimate both the crude and adjusted prevalence rate ratios (PRR), with their corresponding 95% confidence intervals (95% CI). The sampling selection weights were used. The modified Poisson regression model with robust error variance, was preferred to avoid under estimation of standard errors for the estimated risks ratios that is usually the case with logistic regression modeling when the prevalence of the outcome is greater than 10% [20,21]. To identify variables associated with hypertension, all potential risk factor variables for hypertension measured in this study were initially included in the model, and stepwise backward elimination used to remove variables not significantly associated with hypertension. Variables were removed one at a time, starting with those with the largest p-value. A 5% level of statistical significance (α = 0.05) was used to retain variables significantly associated with hypertension. All statistical analyses were performed using STATA version 13.

## Results

### Study sample

Out of the 4900 nationwide sampled subjects, 3987 participated in the NCD risk factor survey, giving a response rate of 81.4%. The remaining 913 sampled subjects either could not be found home on repeated visits by the data collection team, had re-located by the time of the survey, or declined to participate. Of the 3987 that participated in the NCD risk factor survey, 3906 had the three blood pressure measurements and are included this analysis, per the criteria described earlier.

### Characteristics of participants

Of the 3906 participants, 2336 (59.8%) were female, 2849 (72.9%) were rural residents, 2291 (58.7%) were aged between 20 to 39 years, and 1649 (42.4%) had attained at least secondary school education. By region of residence, 744 (19.0%) were from the northern region, 1260 (32.3%) from central, 960 (24.6%) from eastern, and 942 (24.1%) from the western region. The average age of participants was 35.1 (Standard Deviation = 13.1). A summary of selected characteristics of the participants is presented in Table 1.

**Table 1:** Characteristics of participants.

Characteristic		- n-	Summary measure
All participants		3906	100%
Sex:	Females	2336	59.8%
	Males	1570	40.2%
Urban-rural	Rural	2849	72.9%
	Urban	1057	27.1%
Region	Northern	744	19.0%
	Central	1260	32.3%
	Eastern	960	24.6%
	Western	942	24.1%
Age in years:	18–19	336	8.6%
	20–29	1291	33.1%
	30–39	1000	25.6%
	≥ 40	1279	32.7%
	Mean ± SD	3906	35.1 ± 13.0
Level of education attained:	None	639	16.4%
	Primary Sch	1603	41.2%
	Secondary Sch	1287	33.1%
	University or higher	362	9.3%
	Not stated	15	-
BMI^a^ (kg/m^2^)	< 18.5	330	8.9%
	18.5–24.9	2533	68.7%
	25.0–29.9	590	16.0%
	≥ 30.0	236	6.4%
	Not stated	217	-
FPG^b^ (mmol/L)	< 6.1	3500	96.6%
	6.1–6.9	78	2.2%
	≥ 7.0, or on DM Rx^c^	44	1.2%
	Not done	284	-
Alcohol use:	Never	2058	52.7%
	Not using currently	793	20.3%
	Low end user	489	12.5%
	Medium end user	121	3.1%
	High end user	444	11.4%
	Not stated	1	-
Fruit and veg. servings/ day	1–4	3265	87.2%
	≥ 5	480	12.8%
	Not stated	161	-
Met WHO^d^ recommendation for physical activity?	No	221	5.7%
	Yes	3685	94.3%

^a^BMI = Body Mass Index in kilograms per squared meters (kg/m^2^)

^b^FPG = Fasting plasma glucose in millimoles per liter (mmol/L)

^c^DM Rx = Diabetes Mellitus Treatment

^d^WHO = World Health Organization

### Prevalence and distribution of hypertension

Of the 3906 participants, 1033 were classified as hypertensive, giving an overall prevalence of hypertension was 26.4%. Age-standardized to WHO’s 2000–2025 world standard population age structure [22,23], the prevalence was 19.5%. The prevalence was highest in the central region at 28.5%, followed by the eastern region at 26.4%, western region at 26.3%, and the northern region at 23.3%. The prevalence of hypertension was lower in rural residents at 25.8%, compared to urban residents at 28.2%. The prevalence was higher among men at 28.3% compared to females at 25.2%. Hypertension was higher in older age groups, and higher in participants with higher body mass index (BMI). By level of education attained, prevalence was highest among participants who reported that they had not attended any schooling at 30.1%, followed by those with university or higher education at 29.3%, and was lowest among participants with secondary school education at 24.3%. By category of alcohol use, the prevalence of hypertension was highest among high alcohol users at 34.0%, and lowest among participants reporting to have never used alcohol at 24.1%. The distribution of hypertension by the various participant characteristics is summarized in Table 2.

**Table 2:** Distribution of hypertension by population characteristics (un-adjusted).

			Pre-hypertension		Hypertension	
Variable/Category		-n-	# (%)	p-value	# (%)	p-value
	Overall	3906	1441 (36.9%)	n/a	1033 (26.4%)	n/a
Urban-rural	Rural	2849	1050 (36.9%)	0.937	735 (25.8%)	0.132
	Urban	1057	391 (37.0%)		298 (28.9%)	
Region	Northern	744	267 (35.9%)	0.736	173 (23.3%)	0.085
	Central	1260	471 (37.4%)		359 (28.5%)	
	Eastern	960	375 (39.1%)		253 (26.4%)	
	Western	942	328 (34.8%)		248 (26.3%)	
Sex:	Females	2336	779 (33.4%)	< 0.001	589 (25.2%)	0.033
	Males	1570	662 (42.2%)		444 (28.3%)	
Age (yrs):	18–19	336	146 (43.5%)	< 0.001	36 (10.7%)	< 0.001
	20–29	1291	469 (36.3%)		253 (19.6%)	
	30–39	1000	395 (39.5%)		236 (23.6%)	
	40–49	622	254 (40.8%)		197 (31.7%)	
	≥50	657	177 (26.9%)		311 (47.3%)	
Education	None	639	211 (33.0%)	0.152	192 (30.1%)	0.029
	Primary Sch	1603	596 (37.2%)		414 (25.8%)	
	Secondary sch	1287	490 (38.1%)		313 (24.3%)	
	Univ. or higher	362	139 (38.4%)		106 (29.3%)	
Ethnicity	Nyankole	445	157 (35.3%)	0.088	99 (22.3%)	0.156
	Ganda	761	289 (38.0%)		219 (28.8%)	
	Soga	315	120 (38.1%)		77 (24.4%)	
	Nyoro/Toro	253	92 (36.4%)		85 (33.6%)	
	Kiga	254	86 (33.9%)		71 (28.0%)	
	Lugbara/ Madi	245	85 (34.7%)		63 (25.7%)	
	Other	1748	647 (37.1%)		472 (27.0%)	
BMI^a^ (kg/m^2^)	< 25.0	2863	1093 (38.2%)	0.612	702 (24.5%)	< 0.001
	25.0–29.9	590	219 (37.1%)		214 (36.3%)	
	≥ 30.0	236	83 (35.2%)		101 (42.8%)	
FPG^b^ (mmol/L)	< 6.1	3500	1301 (37.2%)	0.397	906 (25.9%)	0.001
	6.1–6.9	78	24 (30.8%)		28 (35.9%)	
	≥ 7.0, or on DM Rx^c^	44	14 (31.8%)		21 (47.7%)	
Met WHO^d^ recommendation for physical activity?	Yes	3685	1374 (37.0%)	0.965	971 (26.1%)	0.070
	No	221	67 (34.9%)		62 (32.3%)	
Tobacco use:	Never	3242	1199 (37.0%)	0.190	832 (25.7%)	0.049
	Stopped	248	105 (42.3%)		79 (31.9%)	
	Current user	336	119 (35.4%)		98 (29.2%)	
Alcohol use:	Never	2058	766 (37.2%)	0.518	496 (24.1%)	0.001
	Currently not using	793	289 (36.4%)		215 (27.1%)	
	Low users	489	183 (37.4%)		139 (28.4%)	
	Medium users	121	51 (42.2%)		32 (26.5%)	
	High users	444	151 (34.0%)		151 (34.0%)	
Dietary salt	Never	1234	434 (35.2%)	0.517	357 (28.9%)	0.137
	Rarely	819	316 (38.6%)		216 (26.4%)	
	Sometimes	1055	398 (37.7%)		269 (25.5%)	
	Often	336	127 (37.8%)		82 (24.4%)	
	Always	461	165 (35.8%)		109 (23.6%)	
Fruit and Veg servings/ day	1–4	3265	1218 (37.3%)	0.865	842 (25.8%)	0.200
	≥ 5	480	181 (37.7%)		137 (28.5%)	

^a^BMI = Body Mass Index in kilograms per squared meters (kg/m^2^)

^b^FPG = Fasting plasma glucose in millimoles per liter (mmol/L)

^c^DM Rx = Diabetes Mellitus Treatment

^d^WHO = World Health Organization

### Awareness of high blood pressure

Of the 1033 participants classified as hypertensive, only 80 (7.7%) reported being aware of their high blood pressure. Bi-variable comparisons showed no significant differences in level of awareness by region (p = 0.245), by sex (p = 0.109), nor by other population characteristic analyzed; except rural-urban residence. Awareness was significantly lower among rural residents (44/735 = 6.0%) compared to urban residents (36/298 = 12.1%) (unadjusted p = 0.001).

### Prevalence and distribution of pre-hypertension

A total of 1441 participants were classified as pre-hypertensive, giving an overall prevalence of pre-hypertension of 36.9%, Table 2. Age-standardized to the World Health Organization’s 2000–2025 world standard population age structure [22,23], the prevalence of pre-hypertension was 25.0%.

### Factors associated with hypertension

The only modifiable factor found to be associated with hypertension in this analysis was higher body mass index (BMI). Compared to participants with BMI less than 25 kg/m^2^, the prevalence of hypertension among participants with BMI between 25 to 29.9 kg/m^2^ was higher with an adjusted PRR = 1.47 [95% CI = 1.29–1.66], and even higher among the obese participants (BMI ≥ 30 kg/m^2^) with an adjusted PRR = 1.67 [95% CI = 1.41–1.99].

The un-modifiable factor found to be associated with hypertension was older age. Compared to participants aged less than 20 years, prevalence of hypertension was higher participants aged 20–29 years with an adjusted PRR of 1.77 [95% CI = 1.19–2.62], and higher among those aged 30–39 years an adjusted PRR of 2.08 [95% CI = 1.40–3.10], even higher among those aged 40–49 years an adjusted PRR of 2.55 [95% CI = 1.71–3.79], and highest among those aged 50 years or older with an adjusted PRR of 3.57 [95% CI = 2.43–5.25].

The other factors we investigated to determine if they were associated with hypertension but did not attain statistical significance included: urban-rural and region of residence, sex, level of education, tobacco use, alcohol use, ethnic group, whether a participant met the WHO recommended weekly level of physical activity or not, frequency of adding salt to food during meal times, and whether a participant took at least five portions of fruits and/or vegetables daily or not. Table 3 gives a summary of the association analysis between the prevalence of hypertension and the various potential risk factors.

**Table 3:** Crude and adjusted prevalence risk ratio estimates for hypertension.

Variable/Category		- n -	# hypertensive (%)	Crude PRR [95% CI]	Adjusted PRR^Ѱ^ [95% CI]
Urban-rural	Rural	2849	735 (25.8%)	1.00	1.00
	Urban	1057	298 (28.2%)	1.02 [0.88–1.18]	1.01 [0.88–1.17]
Region	Northern	744	173 (23.3%)	1.00	1.00
	Central	1260	359 (28.5%)	1.07 [0.88–1.31]	1.05 [0.85–1.28]
	Eastern	960	253 (26.4%)	1.03 [0.83–1.27]	0.99 [0.80–1.22]
	Western	942	248 (26.3%)	1.06 [0.86–1.31]	1.03 [0.84–1.26]
Sex:	Females	2336	589 (25.2%)	1.00	1.00
	Males	1570	444 (28.3%)	1.09 [0.95–1.25]	1.14 [1.00–1.31]
Age (yrs):	18–19	336	36 (10.7%)	1.00	1.00
	20–29	1291	253 (19.6%)	1.83 [1.23–2.74]	1.77 [1.19–2.62]
	30–39	1000	236 (23.6%)	2.29 [1.53–3.42]	2.08 [1.40–3.10]
	40–49	622	197 (31.7%)	2.87 [1.92–4.29]	2.55 [1.71–3.79]
	≥50	657	(311 (47.3%)	4.08 [2.76–6.02]	3.57 [2.43–5.25]
Education	None	639	192 (30.1%)	1.00	1.00
	Primary Sch	1603	414 (25.8%)	0.83 [0.69–0.99]	0.99 [0.83–1.18]
	Secondary sch	1287	313 (24.3%)	0.79 [0.65–0.96]	0.99 [0.82–1.21]
	Univ. or higher	362	106 (29.3%)	0.87 [0.67–1.12]	1.06 [0.82–1.38]
Ethnicity	Nyankole	445	99 (22.3%)	1.00	1.00
	Ganda	761	219 (28.8%)	1.14 [0.88–1.48]	1.10 [0.86–1.42]
	Soga	315	77 (24.4%)	1.27 [0.92–1.73]	1.26 [0.94–1.69]
	Nyoro/Toro	253	82 (33.6%)	1.13 [0.66–1.93]	1.04 [0.62–1.74]
	Kiga	254	71 (28.0%)	1.25 [0.90–1.72]	1.16 [0.84–1.60]
	Lugbara/ Madi	245	63 (25.7%)	1.25 [0.90–1.72]	1.34 [0.97–1.84]
	Other	1633	419 (25.7%)	1.18 [0.94–1.50]	1.17 [0.93–1.47]
BMI (kg/m^2^)	< 25.0	2863	702 (24.5%)	1.00	1.00
	25.0–29.9	590	214 (36.3%)	1.45 [1.24–1.70]	1.46 [1.25–1.71]
	≥ 30.0	236	101 (42.8%)	1.76 [1.42–2.19]	1.60 [1.29–1.99]
FPG^b^ (mmol/L)	< 6.1	3500	906 (25.9%)	1.00	1.00
	6.1–6.9	78	28 (35.9%)	1.20 [0.81–1.78]	1.13 [0.78–1.64]
	≥ 7.0, or on DM Rx^c^	44	21 (47.7%)	1.73 [1.13–2.66]	1.30 [0.86–1.97]
Met WHO^d^ recommendation for physical activity	No	221	70 (31.7%)	1.00	1.00
	Yes	3685	963 (26.1%)	0.82 [0.63–1.06]	0.94 [0.73–1.20]
Tobacco use:	Never	3242	832 (25.7%)	1.00	1.00
	Stopped	248	79 (31.9%)	1.20 [0.96–1.49]	1.09 [0.85–1.39]
	Current user	336	98 (29.2%)	1.29 [1.00–1.66]	1.00 [0.80–1.26]
Alcohol use:	Never	2058	496 (24.1%)	1.00	1.00
	Currently not using	793	215 (27.1%)	1.08 [0.90–1.30]	0.98 [0.82–1.17]
	Low user	489	139 (28.4%)	1.17 [0.89–1.53]	1.04 [0.79–1.37]
	Medium user	121	32 (26.5%)	1.23 [0.85–1.80]	1.26 [0.89–1.79]
	High user	444	151 (34.0%)	1.31 [1.07–1.59]	1.20 [0.99–1.46]
Add salt to food during meals	Never	1234	357 (28.9%)	1.00	1.00
	Rarely	819	216 (26.4%)	0.92 [0.76–1.11]	0.94 [0.78–1.12]
	Sometimes	1055	269 (25.5%)	0.87 [0.73–1.04]	0.93 [0.78–1.10]
	Often	336	82 (24.4%)	0.74 [0.57–0.96]	0.82 [0.63–1.06]
	Always	461	109 (23.6%)	0.84 [0.66–1.08]	0.88 [0.70–1.11]
Fruit and Veg servings/ day	1–4	3265	842 (25.8%)	1.00	1.00
	≥ 5	480	137 (28.5%)	1.03 [0.82–1.28]	1.04 [0.84–1.28]

^Ѱ^Adjusted for: region, sex, age, ethnicity, BMI, and alcohol use

^a^BMI = Body Mass Index

^b^FPG = Fasting plasma glucose

^c^DM Rx = Diabetes Mellitus Treatment

^d^WHO = World Health Organization

## Discussion

Findings from this first nationwide NCD risk factor survey in Uganda reveal a high prevalence of hypertension at 26.5% among adults aged 18 to 64 years, with no significant differences between the four regions of Uganda. Standardized to WHO’s 2000–2025 world standard population age structure [22,23], the age-standardized prevalence was 19.5%. Previous studies in Uganda have reported age-standardized prevalence rates ranging from 19.8% to 30.5% [8–12]. But all these previous studies were conducted small geographical areas and therefore could not provide a nationwide picture of the burden of hypertension.

Unlike other studies in SSA that have found a difference in the prevalence of hypertension between urban and rural dwellers [3,24,25], which are believed to be a reflection of differences in life styles like physical activity and dietary patterns, findings from this survey show that hypertension is equally as high among rural dwellers as it is among urban dwellers. Thus interventions aimed at reducing the burden of hypertension in the country are needed in all regions of the country.

We also found very low levels of awareness among participants with hypertension, with only 7.7% of participants with hypertension aware that they had hypertension. This indicates a high burden of un-diagnosed and un-controlled high blood pressure. This problem has previously been identified by other studies in SSA [3,4,12,26,27], and there seems to be little, or no effort being made to increase the level of awareness regarding hypertension in the general population. This puts the majority of persons with hypertension in Uganda at high risk of hypertension-related NCDs. There is therefore an urgent need to increase detection rates of existing hypertension and provide resources for adequate blood pressure control treatment.

Another important finding was the high prevalence of pre-hypertension, which was above 30% in all the four regions of the country. It is recognised that persons with pre-hypertension are at higher risk of progressing to hypertension [18,28,29]. It has been estimated that approximately 30% of persons with pre-hypertension will become hypertensive within four years [30] if no appropriate interventions are made to arrest or reverse the progress. This is another reason for interventions aimed at increasing regular blood pressure screening to identify persons with risk factors for hypertension. Persons with pre-hypertension should be targeted for risk reduction interventions to prevent progressing to hypertension, which should eventually reduce the risk of hypertension-related NCDs.

In this analysis we were not able to find association between hypertension and some of the factors that have often be identified to be associated with hypertension, for example urban-rural setting[3,25], tobacco use[31], physical activity[31,32] and fruit and vegetable consumption [32]; indicating that there must be other risk factors of fundamental importance for hypertension in the Ugandan setting, other than those analysed here. Thus larger national investigative studies on hypertension are needed to identify other factors that may not have been identified in this national NCD risk factor survey. A possible limitation in our analysis was measurement errors in blood pressure measurements which could result in mis-classification. We however believe that taking three measurements minimized this type of mis-classification.

## Conclusions

The prevalence of hypertension and pre-hypertension is high in Uganda, and only a small percentage of the affected persons are aware of their high blood pressure. Policy makers and stakeholders in the health sector need to institute nationwide population-based strategies to create awareness about hypertension, its main risk factors, as well as its consequences if not controlled, and therefore the need for regular screening.
